# Renal Cell Carcinoma Presenting As Isolated Malignant Pleural Effusion: A Case Report

**DOI:** 10.7759/cureus.73627

**Published:** 2024-11-13

**Authors:** Rahul S Patil, Tejas A Kore, Vikram B Vikhe, Ahsan A Faruqi, Vivek H Lapsiwala

**Affiliations:** 1 General Medicine, Dr. D. Y. Patil Medical College, Hospital and Research Centre, Dr. D. Y. Patil Vidyapeeth (Deemed-to-be-University), Pune, IND

**Keywords:** indwelling pleural catheter (ipc), isolated pleural metastasis, lent score, malignant pleural effusion (mpe), renal cell carcinoma (rcc)

## Abstract

Renal cell carcinoma (RCC) of the clear cell type is the most common form of renal malignancy. Malignant pleural effusion (MPE) as the initial presentation of RCC is very rare. This case report presents a case of RCC that initially manifested as a pleural effusion, without the typical symptoms of flank pain, hematuria, or a palpable abdominal mass. The patient, a 45-year-old male, arrived with complaints of dyspnea and a dry cough, with no notable medical history or risk factors. Imaging revealed a left-sided pleural effusion, which was identified as MPE based on pleural fluid analysis, cytology, and immunohistochemistry. Subsequent investigation revealed a large, solid cystic mass in the left kidney, with a biopsy confirming RCC. Uniquely, the patient exhibited pleural metastasis without involvement of the lung parenchyma, a rare scenario in RCC that is linked to a poor prognosis. This case emphasizes the variable presentation of RCC and the need to consider malignancy in patients with unexplained pleural effusion. An indwelling pleural catheter was placed for the patient considering the poor prognosis; furthermore, he declined to undergo sessions of palliative chemotherapy and was subsequently lost to follow-up.

## Introduction

Renal cell carcinoma (RCC) is responsible for 85% of all kidney tumors, making it the most prevalent type of kidney cancer [1]. Malignant pleural effusion (MPE) is a common cause of exudative pleural effusion among cancer patients, affecting about 15% of these individuals [2]. This case report describes an uncommon clinical manifestation of pleural effusion as an initial presentation of RCC without the clinical symptoms of a palpable abdominal mass, painless hematuria, and flank pain. Genetic risk factors involved in RCC pathophysiology include the polybromo-1 protein gene and the Von Hippel-Lindau gene [1]. Acquired risk factors include obesity, smoking, hypertension, and chronic kidney disease [3]. Histologically, the predominant types of RCC are clear cell, papillary, and chromophobe, arranged in decreasing order of incidence [3]. RCC is most commonly detected by abdominal ultrasonography; in suspected cases of malignancy, computed tomography or magnetic resonance imaging is performed, usually with the use of contrast, as malignant lesions absorb contrast [3]. Although significant advances have been made in understanding the biology of RCC, surgery remains the mainstay of treatment [3]. Pleural metastasis without lung parenchyma involvement occurs in only 4% of patients with clear cell type RCC [4]. The presence of pleural metastasis is associated with the shortest overall survival time of less than 18 months in cases of RCC, indicative of an advanced stage of the disease [4]. This case report highlights the insidious nature and varied clinical presentations of RCC.

## Case presentation

A 45-year-old man with a history of dry cough and progressively worsening dyspnea over the previous seven days came to the ED. He reported no history of fever, hematuria, flank pain, or weight loss and had no known comorbid conditions. The patient, a non-smoker with a history of occasional alcohol use, had the following vitals upon admission: blood pressure of 130/72 mm Hg, pulse rate of 92 per minute, and oxygen saturation of 97% on 2 liters of oxygen. Auscultation of the chest revealed reduced air entry on the left side. Routine blood investigations showed the following values (Table 1).

**Table 1:** Laboratory investigations conducted on the day of admission. SGOT: Serum Glutamic-Oxaloacetic Transaminase; SGPT: Serum Glutamic Pyruvic Transaminase; ALP: Alkaline Phosphatase; PT: Prothrombin Time; INR: International Normalized Ratio.

Parameter	Value	Reference range
Haemoglobin	11.3 g/dL	13.2-16.6 g/dL
Mean corpuscular volume	87.31 fL	78-100 fL
Haematocrit	35.1%	45-74%
Total leucocyte count	7100 cells/cu.mm	4000-11000 cells/cu.mm
Platelet count	304000 /microL	1.5-4.5 /microL
Serum urea	23 mg/dL	15-40 mg/dL
Serum creatinine	1.08 mg/dL	0.6-1.4 mg/dL
Serum uric acid	6.5 mg/dL	2.4 – 6 mg/dL
Serum sodium	140 mmol/L	135-152 mmol/L
Serum Potassium	3.85 mmol/L	3.5-5 mmol/L
Serum chloride	103.4 mmol/L	92-108 mmol/L
Serum ionic calcium	1.19 mmol/L	1.15–1.30 1.30 mmol/L
Serum total bilirubin	1.0 mg/dL	0.1-1.2 mg/dL
Serum direct bilirubin	0.4 mg/dL	0.0-0.5 mg/dL
Serum Indirect bilirubin	0.6 mg/dL	0.0-0.6 mg/dL
SGOT	28 IU/L	0-42 IU/L
SGPT	21 IU/L	0-42 IU/L
Serum ALP	103 IU/L	10-136 IU/L
Serum albumin	4.1 g/dL	3.5-5.5 g/dL
PT/INR	12 seconds / 1.09	9-12 seconds / 0.8-1.2

**Figure 1:**
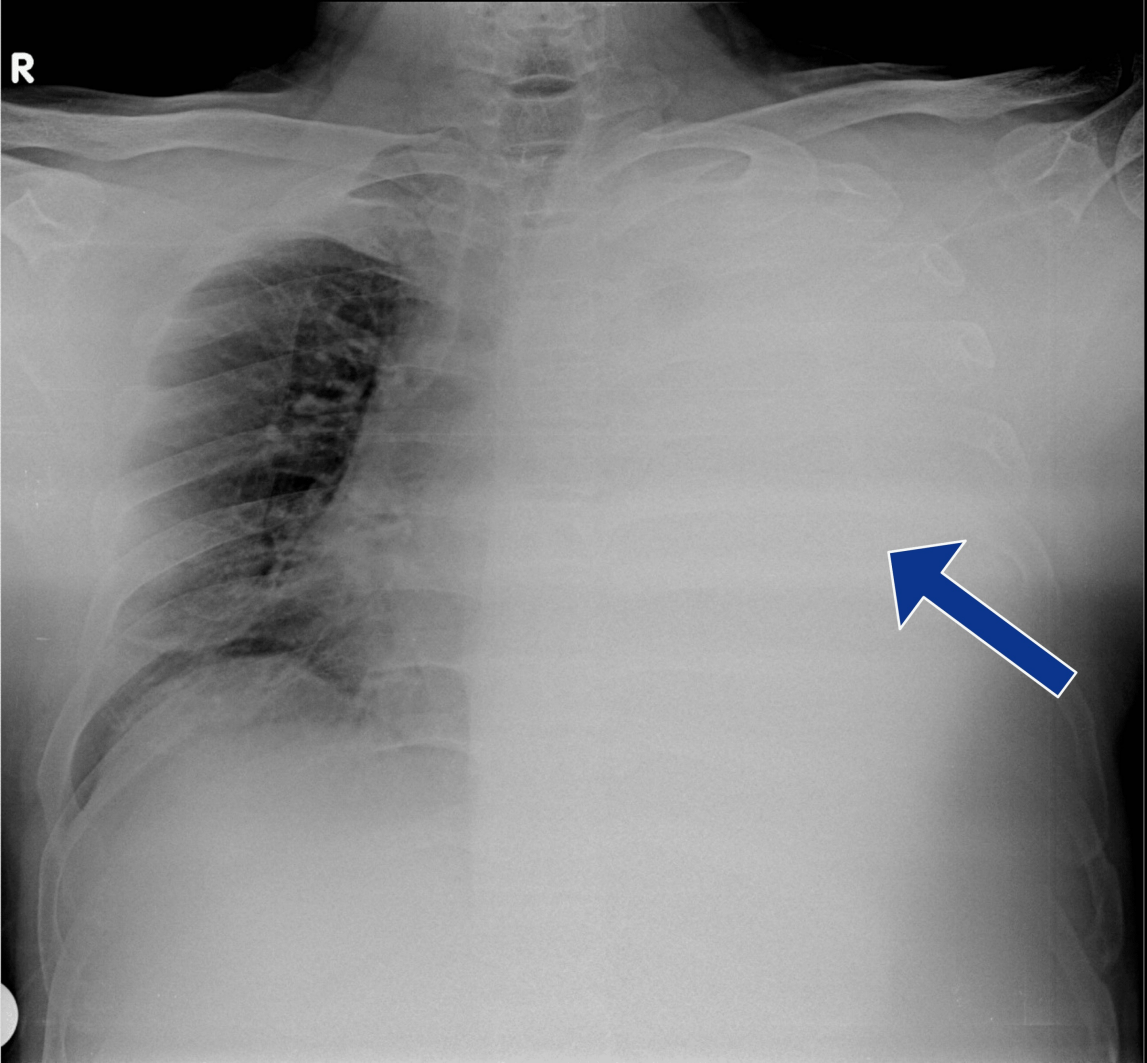
Chest X-ray PA view showing a massive left-sided pleural effusion (blue arrow). PA: Postero-anterior.

**Figure 2:**
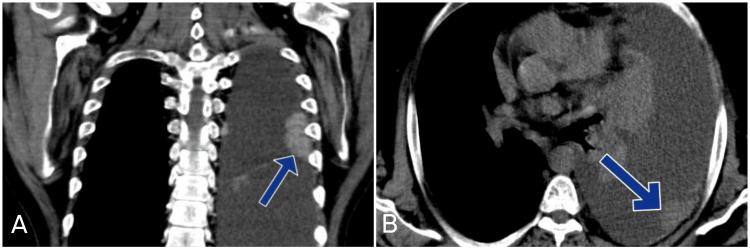
CT scan of the thorax showing soft tissue lesions of the pleura (blue arrows), suggestive of malignancy. (A) Coronal section, (B) Axial section.

**Figure 3:**
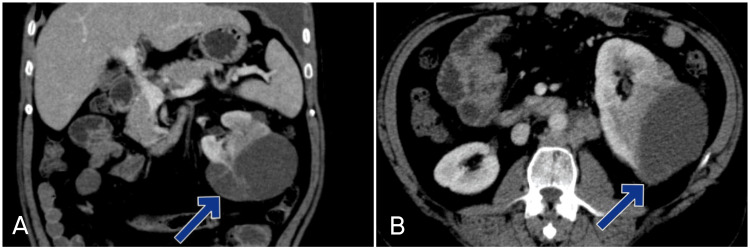
CT scan of the abdomen and pelvis suggestive of a large lobulated solid cystic mass (blue arrows) along the posterior aspect of the left kidney, indicative of malignancy. (A) Coronal section, (B) Axial section.

Laboratory investigations sent on the day of admission were within the normal range, except for mildly decreased hemoglobin and hematocrit (Table 1). A chest radiograph indicated a left-sided pleural effusion (Figure 1), prompting ultrasound-guided therapeutic thoracocentesis, which drained 1.5 liters of serosanguinous fluid. Pleural fluid analysis, based on Light’s criteria, suggested an exudative pleural effusion. Immunohistochemistry of the pleural fluid cell block showed positivity for Vimentin, PAX-8, CAIX, and CK-7 and was negative for calretinin, TTF1, P40, and CK20, confirming the malignant origin of the effusion. On the second day of admission, an abdominal and pelvic ultrasound incidentally showed a hyperechoic solid cystic mass in the left kidney. Further imaging with a computed tomography of the thorax and abdomen confirmed a significant left-sided pleural effusion, multiple enhancing pleural soft tissue lesions (Figure 2), and a large lobulated solid cystic mass along the posterior aspect of the left kidney, indicative of malignancy (Figure 3). A biopsy of the renal mass was performed on the third day of admission, and the patient was discharged on the fourth day. He was told to follow up in a few days until the biopsy report was finalized.

**Figure 4:**
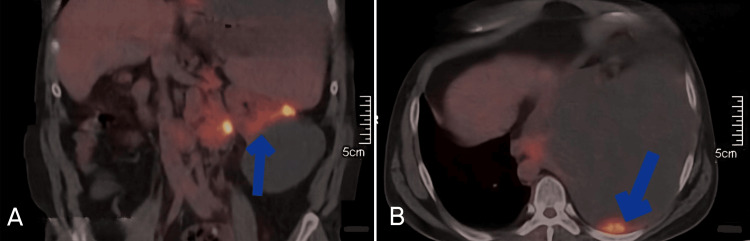
Whole-body PET-CT scan showing: (A) Metabolically active left renal mass (blue arrow), (B) Metabolically active soft tissue mass in the left pleura (blue arrow). PET-CT: Positron emission tomography-computed tomography.

Three days later, the patient returned to the ED with similar symptoms of dyspnea, and recurrent pleural effusion was detected. The renal biopsy results, finalized on the second day of re-admission, confirmed the diagnosis of RCC, clear cell type in the patient. Based on the L- serum lactate dehydrogenase, E- Eastern Cooperative Oncology Group performance score, N- blood neutrophil/lymphocyte ratio, and T- tumor type (LENT score) of 5, the patient was placed in the high-risk category and the decision to place an indwelling pleural catheter (IPC) was made to prevent the need for repeated thoracocentesis [5]. Subsequent whole-body positron emission tomography-computed tomography revealed a metabolically active solid cystic mass in the left kidney, along with retroperitoneal lymph nodes and left pleural deposits (Figure 4). The diagnosis of Stage IV RCC was made, following which the patient and his family were counseled about the poor prognosis and the treatment options available. The patient was not willing to undergo palliative chemotherapy sessions and hence was given supportive treatment with Injection Tramadol 50 milligrams intravenously three times a day for chest pain and prophylactic antibiotic Injection Ceftriaxone 1 gram intravenously twice daily post-IPC placement. He was discharged on the fourth day of re-admission and told to follow up after a week, but was subsequently lost to follow-up.

## Discussion

RCC, also known as the internist’s tumor, presents as the clinical triad of flank pain, hematuria, and palpable abdominal mass in only 10% of RCC cases. A Japanese study found that only 12% of RCC patients had pleural metastases; however, no case had solitary pleural metastases, making this an unusual occurrence [6]. Pleural effusion is the pathological accumulation of fluid in the pleural space. The larger the spatial extent of the pleural effusion, especially if it is unilateral, the more likely it is caused by malignancy [7]. To the best of our knowledge, only 14 case reports of RCC presenting as pleural effusion upon admission have been documented. Wasifuddin M et al. conducted a meta-analysis of the existing 13 cases, which yielded the following conclusions: one instance had no metastases to the lung or pleura, five case reports mentioned patients having metastases to both the lung and pleura, six case reports mentioned patients having isolated pleural metastases, and one case report did not specify the involvement of the lung or pleura [8].

Clear cell RCC most commonly metastasizes to the lung, liver, bone, and lymph nodes [4]. The least common sites of RCC metastases are the pleura, peritoneum, pancreas, spleen, thyroid, and bowel [4]. The median survival time for patients with pleural metastases is 15.6 months. Previously, patients with metastatic RCC were treated with immunosuppressive agents like IL-2 and INFα. Nowadays, agents targeting biological processes are being used for metastatic RCC, such as drugs targeting vascular endothelial growth factor, platelet-derived growth factor, and mammalian target of rapamycin [3]. Tyrosine kinase inhibitors are also used in the advanced stages of RCC as a first-line drug.

The LENT score helps in predicting the survival of patients with malignant pleural effusion [5]. Based on the Eastern Cooperative Oncology Group performance score, levels of pleural fluid lactate dehydrogenase, tumor type, and serum neutrophil to lymphocyte ratio, the LENT score classifies patients into three risk groups: low, moderate, and high, with median survival times of 319 days, 130 days, and 44 days, respectively [2]. The management of recurrent malignant pleural effusion should be based on the LENT score; placement of an IPC is more cost-effective and less invasive for patients with a life expectancy of less than 3 months, whereas talc pleurodesis is performed in patients with a higher life expectancy [2]. As our patient had a LENT score of 5, he was placed in the high-risk category, and an IPC was placed for symptomatic relief. Similar cases have been reported by Wasifuddin M et al. [8], Hutchinson AH et al. [9], and Kataoka M et al. [10].

## Conclusions

This case illustrates the rare presentation of RCC as a malignant pleural effusion, highlighting the significance of considering RCC as a differential diagnosis in cases of unilateral MPE as the initial presentation. The absence of typical RCC symptoms, such as a palpable abdominal mass, flank pain, and hematuria, underscores the diverse ways in which this cancer can manifest, potentially leading to diagnostic delays. The unusual finding of pleural metastasis without lung parenchymal involvement in this case is particularly significant, as it generally indicates a more advanced stage of RCC with a poor prognosis.

This case emphasizes the need for thorough diagnostic evaluations in patients presenting with pleural effusion, even when other common symptoms of cancer are absent. The rarity of isolated pleural metastasis in RCC, as demonstrated in this instance, further underscores the necessity of considering a broad range of differential diagnoses to ensure timely and effective treatment.
